# Highly Promising 2D/1D BP‐C/CNT Bionic Opto‐Olfactory Co‐Sensory Artificial Synapses for Multisensory Integration

**DOI:** 10.1002/advs.202403665

**Published:** 2024-06-03

**Authors:** Liyan Dong, Baojing Xue, Guodong Wei, Shuai Yuan, Mi Chen, Yue Liu, Ying Su, Yong Niu, Bingshe Xu, Pan Wang

**Affiliations:** ^1^ Xi 'an Key Laboratory of Compound Semiconductor Materials and Devices School of Physics & Information Science Shaanxi University of Science and Technology Xi'an 710021 P. R. China; ^2^ Shanxi‐Zheda Institute of Advanced Materials and Chemical Engineering Taiyuan 030024 P. R. China

**Keywords:** artificial synaptic, biological bionic, BP‐C, carbon‐doped black phosphorus, opto‐olfactory perception, passivation, sympathy

## Abstract

The development of high‐performance artificial synaptic neuromorphic devices poses a significant challenge in the creation of biomimetic sensing neural systems that seamlessly integrate both sensory and computational functionalities. In pursuit of this objective, promising bionic opto‐olfactory co‐sensory artificial synapse devices are constructed utilizing the BP‐C/CNT (2D/1D) hybrid filter membrane as the resistive layer. Experimental results demonstrated that the devices seamlessly integrated the light modulation, gas detection, and biological synaptic functions into a single device while addressing the challenge with separating artificial synaptic devices from sensors. These devices offered the following advantages: 1) Simulating visual synapses, they can effectively replicate fundamental synaptic functions under both electrical and optical stimulation. 2) By emulating olfactory synapse responses to specific gases, they can achieve ultra‐low detection limits and rapid identification of ethanol and acetone gases. 3) They enable photo‐olfactory co‐sensing simulations that mimic synaptic function under light‐modulated pulse conditions in distinct gas environments, facilitating the study of synaptic learning rules and Pavlovian responses. This work provides a pioneering approach for exploring highly stable 2D BP‐based optoelectronics and advancing the development of biomimetic neural systems.

## Graphical abstract

In this study, high‐stability carbon‐doped, and carbon passivation black phosphorus‐based bionic opto‐olfactory co‐sensory artificial synapses are successfully developed, achieving light modulation, gas detection, and biological synaptic functions.

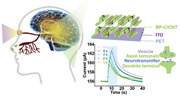

## 1 Introduction

The human brain not only processes single‐modal sensory inputs but also integrates multi‐sensory information through specialized neurons in the primary sensory areas, thereby eliciting better and faster responses in the human nervous system.^[^ ^1^, ^2^ ^]^ In light of this, the development of highly promising neuromorphic devices that mimic the human sensory organs (eyes, ears, nose, tongue, and skin) for perceiving diverse information,^[^ ^3^ ^]^ is crucial for achieving neural morphology, perception, computation and bridging the gap between artificial and natural intelligence.^[^ ^4^, ^5^, ^6^, ^7^ ^]^ To date, artificial biomimetic sensory systems have been developed toward intelligent artificial synapse systems capable of device‐level perception, filtering, computing, and memorization.^[^ ^8^, ^9^, ^10^, ^11^ ^]^ Among them, memristor‐derived artificial synapse devices possess comparative advantages in complex tasks such as learning, classification, recognition, sensing, information storage, and neural network simulation.^[^ ^12^, ^13^, ^14^ ^]^

Perception serves as the interface and channel through which the human brain interacts with the environment to obtain sensory information, predominantly facilitated by the visual and olfactory systems as crucial sensory platforms. In recent years, there has been a growing research interest in employing light for photoelectric neural synaptic devices to simulate human vision,^[^ ^15^, ^16^, ^17^, ^18^ ^]^ given that over 70% of human information is derived from the visual channel. Zhu Chenguang et al. proposed a low‐power artificial photonic synapse based on the black phosphorus (BP)/CdS heterostructure, providing novel insights into designing high‐efficiency artificial visual synapses and high‐performance visual systems.^[^ ^19^ ^]^ Additionally, alongside visual synaptic devices, olfactory synaptic devices can possess significant potential for recognizing numerous odor molecules, enabling artificial intelligence (AI) to resemble humans in perceiving their surroundings, evaluating food quality, and identifying potential hazards.^[^ ^20^, ^21^ ^]^ However, delayed advancements in olfactory sensing algorithm technology and limited application scope along with poor recognition accuracy issues still prevalent at early stages of development hinder progress in this field.^[^ ^22^ ^]^ Deng et al.^[^ ^22^ ^]^ proposed a flexible and biomimetic olfactory synapse based on an organic electrochemical transistor coupled with a breath‐figure derived porous solid polymer electrolyte, demonstrating the potential for advanced sensory applications. In 2019, Iwata et al. reported a novel memristor‐based olfactory sensor array that successfully between acetone and ethanol.^[^ ^23^ ^]^

Enabling multiple senses in a single device enhance AI's ability to perceive and comprehend the surrounding environment more accurately, thereby greatly improving information processing efficiency and intelligence. Notably, remarkable advancements have been achieved in the perception of visual, auditory, and tactile information in the field of artificial nervous systems.^[^ ^5^, ^24^, ^25^, ^26^ ^]^ Liu et al.^[^ ^24^ ^]^ fabricated a nanowire‐channel intrinsically stretchable neuromorphic transistor capable of perceiving both tactile and visual stimuli while emulating neuromorphic processing capabilities. You et al.^[^ ^27^ ^]^ constructed an artificial nervous system for simulating perception and synaptic plasticity. Sun et al.^[^ ^28^ ^]^ reported an artificial reflex arc that sensed/processed visual and tactile information using a self‐powered optoelectronic perovskite artificial synapse, enabling controlled muscular actions in response to environmental stimuli. These reports highlight the significance of developing neural synaptic devices that can simulate multiple sensory organs, realize biological synaptic signal processing, and exhibit neurobiological behaviors to achieve neural morphological perception and computation by simulating multi‐sensory neuron responses, thus bridging the gap between artificial intelligence and natural intelligence.

2D layered BP exhibits numerous extraordinary photoelectricity characteristics, including the thickness‐dependent tunable bandgap (0.3–2.0 eV), in‐plane anisotropy, high carrier mobility, and large absorption coefficient, making it an ideal candidate for the development of highly promising optoelectronic and olfactory synaptic devices.^[^ ^29^ ^]^ However, the inherent instability of BP materials in the environment severely hinders their further development in the field of synaptic devices. Several approaches have been developed to overcome this limitation, among which the in situ doping strategy has garnered more attention due to its ability to modulate both electronic structure and physicochemical properties without compromising BP's unique 2D structure. The electronegativity similarity between carbon atom (C) and phosphorus atom (P) enables easy adsorption of C atom into interstitial spaces or atomic substitution with P atom. Additionally, forming van der Waals heterostructures by stacking 2D BP with other low‐dimensional carbon nanomaterials presents another promising strategy for enhancing BP stability. It has been reported that extended pairs of BP can act as electron donors and effectively bind and interact with graphene (or graphene oxide), facilitating the formation of P−C or P−O−C bonds. This interaction enhances BP stability while promoting cost‐effective hybrid materials development based on BP.^[^ ^30^ ^]^ In our recent studies conducted in 2022, we successfully fabricated robust and low‐energy artificial synapses using BP by leveraging its interplay with graphene.^[^ ^21^ ^]^

In this study, the highly stable carbon‐doped BP (BP‐C) was successfully developed through in situ carbon doping and synchronous passivation strategies using an improved chemical vapor transport approach with the addition of a solid carbon source. All characterization results consistently demonstrated that C‐doping effectively enhanced the stability of BP, which is in agreement with first‐principle calculations. Based on these findings, we fabricated bionic opto‐olfactory co‐sensory artificial synapse devices by employing a BP‐C/CNT (2D/1D) heterostructure‐based filter membrane as the functional active layer. The devices possess the ability to mimic characteristic features and functionalities of multisensory neurons by simultaneously integrating light modulation, gas sensing, and synaptic functions within a single device. The experimental results further demonstrated that the device not only emulated fundamental synaptic functions under electrical and optical stimuli but also facilitated rapid detection of various gas concentrations, including ethanol and acetone. Moreover, it effectively simulated synaptic functions under different gas conditions, successfully emulating double‐pulse potentiation, facilitating the transition from short‐term memory (STM) to long‐term memory (LTM) memory, and accurately simulating synaptic learning rules. Furthermore, the device successfully realized Pavlovian simulation for learning functions. Obviously, this contribution could pave the way for the widespread application of antioxidant 2D BP materials in optoelectronics while providing a novel approach for studying opto‐olfactory synapse devices—a significant step toward flexible and neuromorphic electronics compatible with the human body.

## 2 Results and Discussion

### 2.1 Preparation, Characterization, and First‐Principles Calculations of BP‐C Materials

**Figure 1:**
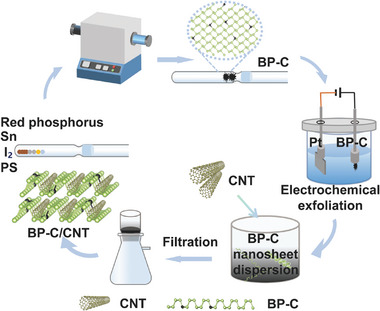
Schematic diagram of the sequential steps involved in the preparation of BP‐C hybrid composites and 2D/1D BP‐C/CNT heterostructure‐based filter membrane.

The achievement of one‐step in situ synthesis of BP‐C hybrid composites, incorporating carbon element doping and synchronous carbon passivation, was successfully accomplished utilizing an improved chemical vapor transport method by adding polystyrene as a solid carbon source (**Figure** **1**). This fabrication method not only provides a promising BP in situ doping technology for developing highly stable BP materials from the beginning but also offers a solution to the challenges of follow‐up BP exfoliation and oxidation. By employing the electrochemical cathodic exfoliation technique, large‐sized 2D BP‐C nanosheets with dimensions ranging from ≈20–100 µm (as depicted in Figure S1a,b, Supporting Information) were successfully obtained from bulk BP‐C materials. Compared with the exfoliation process of carbon‐doped BP and undoped BP, it was observed that the exfoliation process for carbon‐doped BP can be completed more efficiently. As illustrated in Figure S2 (Supporting Information), when most bulk BP‐C was exfoliated into 2D nanosheets, the time for achieving similar exfoliation degree is remarkably shortened to only 10–15 s under a voltage of 10 V compared to ≈30–40 s needed for undoped BP. Obviously, this notable improvement in exfoliation speed can be attributed to the incorporation of carbon element doping in bulk BP. Subsequently, the resulting 2D BP‐C nanosheets were mixed with 1D carbon nanotubes (CNTs) through an ultrasonication process for 10 min to construct a stable and efficient BP‐C/CNT (2D/1D) heterostructure consisting of coupled P─C and C─C bonds between the two components (BP‐C/CNTs). It is anticipated that this unique structure combining 2D nanosheets with 1D tubes can possess exceptional conductivity, high stability, and intrinsic activity, thus making it highly promising for diverse applications.

**Figure 2:**
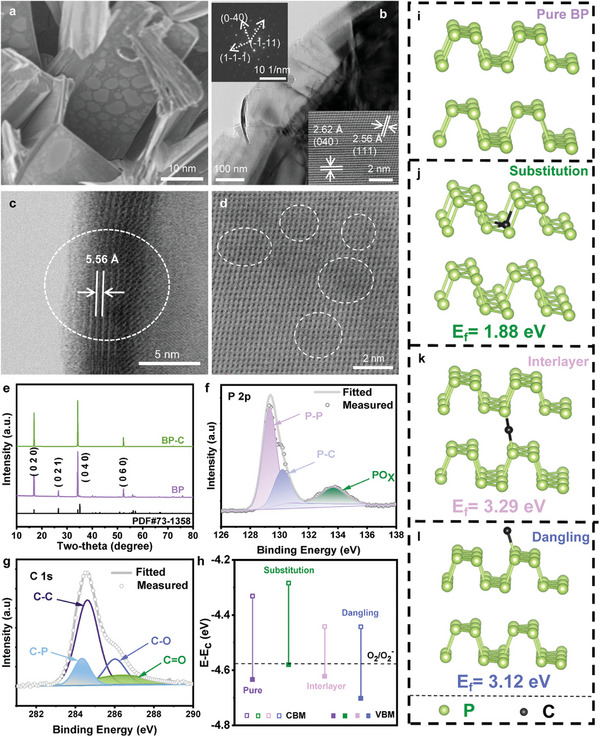
a) Typical SEM image of bulk BP‐C. b) TEM, HRTEM, and SAED images of exfoliated 2D BP‐C nanosheets. c) HRTEM image of cross‐section of BP‐C. d) Atomic phase of BP‐C under aberration‐corrected electron microscope. e) XRD patterns of bulk BP and BP‐C. f,g) XPS spectrum of BP‐C. h) The valence band maximum (VBM) and conduction band minimum (CBM) of pure BP and C‐doped BP with respect to vacuum energy (*E*_c_). The dashed line represents the relative position of the redox potential of O_2_/O_2_^−^. The model configurations of i) the ideal bilayer BP and with j) one P atom replaced by one C atom, k) one interlayer C atom, and l) one surface dangling C atom, respectively.

The microstructure analysis and surface characterization of both unexfoliated bulk BP‐C materials and pure BP materials were conducted using a scanning electron microscope (SEM). **Figures** **2**a and S3a (Supporting Information) show the typical SEM images of the BP and BP‐C samples, respectively, showcasing their exceptional crystallinity with well‐defined rectangular grain morphology. Notably, a remarkable transformation in the surface morphology of the bulk BP‐C can be observed after the carbon doping process, wherein a substantial portion of the surface becomes coated with wrinkled and bubble‐like carbon texture. The alteration in morphology further emphasizes the successful incorporation of carbon into the BP structure. To gain a deeper insight into the elemental composition of the sample, energy spectroscopy was performed on a randomly selected area of the material, revealing multiple elements including carbon (C), phosphorus (P), tin (Sn), iodine (I), and others (Figure S4, Supporting Information). To further validate the quality of the generated crystal samples, transmission electron microscopy (TEM) characterizations were also conducted on both materials. As depicted in Figure 2b; Figures S3b and S9 (Supporting Information), the obtained TEM results can provide compelling evidence of the high quality and purity of the crystal. The high‐resolution transmission electron microscopy (HRTEM) images exhibit distinct lattice fringes, with the (020) crystal face of BP shown in Figure S3b (Supporting Information) and the (111) and (040) faces of BP‐C depicted in Figure 2b. Furthermore, a comprehensive analysis utilizing spherical aberration electron microscopy uncovered the influence of carbon atoms on the lattice structure and interlayer spacing of BP crystal structure for BP‐C materials. Figure 2c presents a side‐view HRTEM image demonstrating an interlayer spacing from 5.216 Å of BP to 5.56 Å for exfoliated BP‐C nanosheets. This finding provides compelling evidence regarding the impact of carbon doping on the structural properties of BP at the atomic level in BP, shedding light on their interplay between carbon and BP. In Figure 2d, significant atomic‐scale lattice distortions can be observed in the single‐layer 2D BP‐C nanosheet, indicating a significant impact from in situ carbon doping on the structure of BP. To conduct a comprehensive analysis, X‐ray diffraction (XRD) was performed to determine crystalline nature as well (Figure 2e). For the BP nanosheets, distinct peaks at 16.8°, 26.4°, 34.2°, and 52.3° correspond to the crystal planes of (020), (021), (040), and (060), respectively are observed in the XRD pattern. However, a significant difference is noticed in the XRD pattern of the BP‐C material compared to BP. Specifically, there is an absence of the (021) crystal plane peak in the XRD pattern of BP‐C material, indicating that carbon incorporation into the BP lattice could induce alterations in crystal plane arrangement and orientation leading to a distinct XRD pattern. To gain further insight into how carbon doping influences structural properties of BP, we conducted first‐principles calculations on the bilayer BP doped with carbon atoms. Figure 2 illustrates three potential configurations that were considered: substitution of one P atom with one C atom (Figure 2j), intercalation of one C atom into bilayer BP (Figure 2k), and absorption of one C atom on bilayer BP surface as the dangling form (Figure 2l). The formation energies of the three structures are calculated as follows: replacement −1.88 eV, intercalation −3.29 eV, and absorption −3.12 eV, respectively (see Table S1, Supporting Information). Computational results suggest that energetically C atoms tend to substitute for P atoms in doped BP regardless of configuration employed here worth mentioning that direct band structure transforms into indirect band structure upon doping with C atoms (see Figure S5, Supporting Information).

The surface composition and chemical states of the samples were evaluated using X‐ray photoelectron spectroscopy (XPS) was used to evaluate. The results demonstrate the presence of three elements sample: carbon (C), phosphorus (P), and oxygen (O) on the surface of the BP‐C. This finding further confirms the successful incorporation of carbon into the BP structure during synthesis. In Figure 2f, the high‐resolution P 2p spectrum of BP‐C shows a strong peak at 129.3 eV attributed to the P─P bond of 2p_3/2_ and 2p_1/2_, while a peak at 130.3 eV is assigned to the P─C bond. Additionally, a small peak at 133.7 eV corresponding to PO_x_ indicates unavoidable oxidation of exfoliated 2D BP‐C nanosheets after exposure to air for more than one month. The C 1s spectrum (Figure 2g) further confirms the formation of P─C bonds at 284.0 eV, indicating that in situ carbon doping enhances overall antioxidation performance, and achieve passivation in BP material. These XPS findings provide insights into surface composition and chemical states of the BP‐C material, highlighting successful incorporation of carbon and its positive impact on the antioxidation performance.

To further validate the stability and antioxidative performance of the BP‐C material, we conduct several observations on the surface morphology of both BP and BP‐C samples under ambient conditions. This experimental design aimed to assess the degradation behaviors of the materials over time, as illustrated in Figure S6 (Supporting Information). Figure S6a–c (Supporting Information) depicts optical microscope images of original BP after one day, three months, and one year, respectively. Figure S6d–f (Supporting Information) displays optical microscope images of BP‐C after one day, three months, and one year, respectively. From these images captured by the optical microscope, it is evident that undoped BP starts to decompose after just one day with significant decomposition observed on its surface after 3 months. In contrast, BP‐C exhibits a noticeably slower oxidation and decomposition behavior compared to pure BP with only slight decomposition observed after 3 months. These comparative optical microscope images provide compelling evidence for the improved antioxidative performance of the BP‐C material. To elucidate the mechanism underlying the enhanced stability of carbon doping BP, the valence band maximum (VBM) and conduction band minimum (CBM) of pure BP and carbon doping BP with respect to vacuum energy (*E*_c_) were further calculated (Figure 2h; Figure S5, Supporting Information). In the case of pure BP and interlayer/dangling form carbon doping BP, the redox potential of O_2_/O_2_^−^ is located within the bandgap, facilitating photo‐generated electrons transfer from the conduction band of BP to surface O_2_ molecules, resulting in O_2_^−^ generation. This process serves as a crucial indicator for activating the degradation process of BP. However, when C is substituted into BP atoms leading to substitution carbon doping in BP with higher VBM than that of redox potential of O_2_/O_2_^−^, it causes the redox potential of O_2_/O_2_^−^ to lie outside the bandgap_._ Moreover, substitution for P atom represents the main form of C doping in BP due to its lowest formation energy among various configurations of C dopants. Consequently, carbon doping BP exhibits reduced generation of O_2_^−^ anions and effectively suppresses oxidation process, thereby further enhancing its stability.

### 2.2 Design and Fabrication of the Device

In biology, sensory neurons convert external stimulation signals into nerve impulses. The 2D/1D heterostructure possesses unique optoelectronic and electronic transport properties, as well as controllability and stability. The high surface activity of BP‐C material arises from the substitution of carbon atoms for phosphorus atoms and the presence of lone pair electrons carried by 2D BP itself. By utilizing the synergistic effect between 1D carbon nanotubes (CNTs) and 2D BP‐C nanosheets, the hybrid heterostructures of 2D/1D BP‐C/CNT demonstrate enhanced electrical conductivity and superior electrophilicity. In this configuration, CNTs serve as a bridge for stabilizing the structure of 2D BP, facilitating stronger interlayer interactions and overall material stability. Additionally, due to their inherent 1D nature, CNTs efficiently transfer stress while providing additional support to form functional film based on BP‐C, thereby reducing crack formation and material failure. To gain insight into its structural composition, characterization was performed on the BP‐C/CNT (2D/1D) material. The SEM images in Figure S10a,b (Supporting Information) demonstrate the overall curved morphology of carbon nanotubes and their facile interaction with the surrounding BP‐C materials to form the BP/CNT heterostructure. Additionally, Figures S7a and S10c,d (Supporting Information) exhibit SEM characterization images of BP‐C/CNT membranes, revealing the presence of a layered structure consisting of BP‐C and tubular carbon nanotubes within the heterostructures. These 2D BP‐CNSs and 1D carbon nanotubes are interconnected, resulting in a loose and porous structure, which is conducive to fast electron transfer and gas adsorption. The TEM image in Figure S12 (Supporting Information) illustrates the BP‐C/CNT (2D/1D) heterogeneous structure, where CNTs encapsulate BPNSs, thereby forming a corresponding heterogeneous interface. Through this interface, electrons are transferred from BP NSs to carbon nanotubes, altering the electronic structure of the interface and resulting in a highly conductive and electrophilic BP/CNT heterostructure. XRD analysis (Figure S7b, Supporting Information) demonstrates that the prepared samples consist of both the BP‐C material and CNTs. The peaks at 16.8°, 34.2°, and 52.3° correspond to (020), (040), and (060) planes of BP‐C nanosheets, respectively, while a peak at ≈26.3° in CNTs can be attributed to the (002) plane of the hexagonal graphite structure.

**Figure 3:**
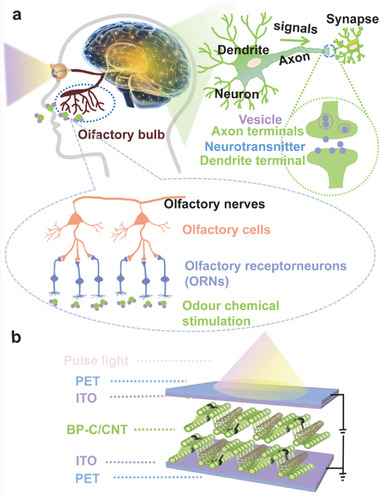
Multisensory integration. a) A Schematic representation of multisensory integration of visual and olfactory information within the biological nervous system and neural synapses. b) Schematic diagram of the device structure.

Pulses are then transmitted to the next layer of neurons through synapses in the form of action potentials.^[^ ^24^ ^]^ Both the visual and olfactory sensory systems play a significant role in brain function. The visual system facilitates the transmission and processing of visual information through intricate visual synapses, enabling the brain to comprehend and interpret visual stimuli. Similarly, the olfactory system operates by transmitting information via olfactory synapses located within the olfactory pathway, serving as vital conduits for processing olfactory information. **Figure** **3**a provides a schematic representation to enhance understanding of these sensory systems in living organisms. Ultimately, the information is processed by synapses in the brain, contributing to overall cognitive functioning.

The BP‐C/CNT (2D/1D) heterostructure can serve as the functional active layer in bionic artificial synapse devices. When the BP‐C/CNT hybrid film is used to fabricate synapse devices, it requires that the film can be relatively stable and exhibit good performance. In order to verify the feasibility of proposed BP‐C/CNT‐based synaptic devices, we fabricated artificial synapse devices with a PET/ITO/BP‐C/CNT/ITO/PET structure to achieve synaptic functionality (Figure 3b). As shown in Figure 3b, this synaptic device structure with a typical sandwich configuration closely resembles the biological synapse structure. The PET/ITO top electrode corresponds to the presynaptic membrane, while the ITO/PET at the bottom corresponds to the postsynaptic membrane, and the BP‐C/CNT functional layer serves as an intermediary for synaptic transmission. Neurotransmitters are initially generated by stimulation signals at the presynaptic membrane and then interact with receptors on the postsynaptic membrane, leading to corresponding action potentials in postsynaptic membrane that mimic synaptic behavior. Depending on stimulus form, these action potentials correspond to either potentiation or inhibition of synapses. In the artificial synapse device, PET/ITO electrodes function as the presynaptic membrane for receiving stimulation signals, where electrons are injected from the PET/ITO side resulting in neurotransmitter release. These electrons traverse through the BP‐C/CNT layer before reaching graphite bottom electrode, thereby generating a current that can be regarded as a postsynaptic current.

### 2.3 Synaptic Plasticity of the Device in Light and Electrical Stimulation States

**Figure 4:**
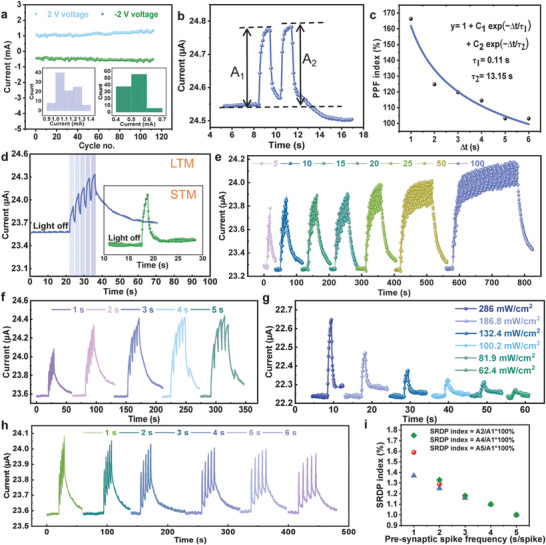
a) Current variation of the synaptic device over 100 I‐V cycles. b) Simulation test of light pulse‐pair potentiation in the device (365 nm, 286 mW cm^−2^, pulse width 1 s, pulse interval 1 s). c) Ratio of paired‐pulse facilitation index to the time interval between two pulses. d) Transition of the device to LTM mode after five consecutive light pulses at a bias of 1 V (the inset shows the STM mode after a single light pulse). e) SNDP test at different numbers of light pulses. f) SDDP test at the different light durations. g) Peak power‐dependent plasticity (PPDP). h) SRDP test at different pulse rates. i) SRDP index.

To evaluate the synaptic function simulation of the BP‐C/CNT device, cyclic voltage (CV) tests are performed, as shown in **Figure** **4**a. By subjecting the device to a hundred cycles of voltage testing, a stable testing curve can be obtained, ensuring the reliability and stability of devices based on this architecture. These results confirm that BP‐C/CNT devices exhibit bidirectional synaptic plasticity, thereby validating their potential as synaptic devices and facilitating the development of high‐performance artificial synapse devices. Synaptic plasticity refers to the characteristic of adjustable synaptic connection strength between neural cells. It mainly includes short‐term synaptic plasticity and long‐term synaptic plasticity. The rich synaptic plasticity enables synapses to perform various functions in information processing, making the simulation of biological synaptic plasticity crucial. Short‐term synaptic plasticity is a fundamental manifestation of synaptic plasticity and plays a crucial role in achieving normal functioning of the nervous system. The application of presynaptic pulses can trigger excitatory postsynaptic currents (EPSCs). Paired‐pulse facilitation (PPF) function serves as the primary mechanism in spike‐dependent plasticity, exerting a significant influence on the recognition and decoding of transient information by the nervous system. Two consecutive light pulses (365 nm, 286 mW cm^−2^, pulse width 1 s, pulse interval 1 s) are applied, resulting in an EPSC triggered by the second pulse that is significantly higher than that generated by the first pulse (Figure 4b). Typically, the facilitatory effect is quantified using the paired‐pulse facilitation (PPF) index (A_2_/A_1_ × 100%), where *A*_1_ and *A*_2_ represent the amplitude of the response currents to the first and second pulses, respectively. The output amplitude of PPF induced by the second light pulse depends on the interval between the signals. As the time interval (Δt) between the two consecutive pulses increases, both facilitatory effects weaken, and synaptic weight changes decrease accordingly (Figure 4c). These experimental PPF indices can be fitted with a double‐exponential function given by y = y_0_ + C_1_exp(−∆t/τ_1_) + C_2_exp(−∆t/τ_2_). The fitting results reveal τ_1_ to be 0.11 s and τ_2_ to be 13.15 s.

Frequent synaptic stimulation can induce the formation of synaptic memory and facilitate the transition from STM to LTM, which represents a crucial function of biological synapses.^[^ ^7^ ^]^ The most straightforward approach to achieving LTM is through continuous training that provides sustained synaptic stimulation. Figure 4d depicts the current‐time response, showing a single current peak resulting from a brief light pulse applied to the device. Once the light is deactivated, the current decays gradually instead of an immediate return to its initial level exhibiting persistent photocurrent. Within 5 seconds, the current recovers back to its initial state, indicating STM behavior. In contrast, when subjected to five consecutive light pulses with random durations, each pulse's peak current (Figure 4d) exhibits significantly slower decay compared to a single pulse. Therefore, multiple light pulses lead to the transition from STM to LTM in this context and highlight BP‐C/CNT as potential optical synaptic memory for neuromorphic computing applications.

The level of memory in the human brain is correlated with the intensity of learning, and optoelectronic synaptic devices can gradually transition from short‐term plasticity (STP) to long‐term plasticity (LTP) by adjusting optical stimulation parameters, such as power density, pulse width, number, and frequency. This gradually adjustment enables a progressive increase in synaptic weight from weak to strong. Simultaneously, as STP transitions to LTP, the decay time (τ) and steady‐state current of the postsynaptic current (PSC) also increase. Increasing the number of light pulses (365 nm, 286 mW cm^−2^, pulse width 1 s, pulse interval 1 s) leads to an elevation in EPSC values generated by the synapse, suggesting an enhanced memory level response to greater stimulation. The decay curve of the artificial synapse's current exhibits a similar pattern to the forgetting curve observed in the human brain. Memory duration significantly prolongs as evidenced by Figure 4e when there is an increase in consecutive peaks up to 100. From Figure 4f, it can be clearly seen that the EPSC values increase when the device durations of light pulses are used for device stimulation (365 nm, 286 mW cm^−2^, pulse width ranging from 1 to 5 s). This behavior is known as spike duration‐dependent plasticity (SDDP). In addition, high‐frequency stimulation elicits notable responsiveness from the synaptic device. Peak power‐dependent plasticity (PPDP) represents a transient modulation of synaptic activation response under varying stimulus intensities. When light pulses with different optical power densities (ranging from 62.4 to 286 mW cm^−2^) are irradiated onto the BP‐C/CNT artificial synapse device, the EPSC increases with the increase in optical power density (from 22.27 to 22.65 µA) (Figure 4g).

Spike rate‐dependent plasticity (SRDP) refers to a sustained modulation of synaptic responsiveness, describing the conductance changes of analog memristors based on specific spike rates. Additionally, EPSC can increase with higher presynaptic stimulation frequencies, aligning with the fundamental characteristics of biological synapses. By manipulating the time intervals between stimuli, the transition from STM to LTM can be observed. When subjecting the device to continuous light pulses at different pulse intervals (365 nm, 286 mW cm^−2^, pulse width of 1 s, pulse intervals ranging from 1 to 6 s) for 5 times, EPSC gradually decreases over time (Figure 4h). Notably, an increased presynaptic membrane pulse rate leads to a greater current increment in the postsynaptic membrane and strengthens the connection between both membranes. As expressed in Figure 4i, shorter time intervals result in higher conductivity increments. Therefore, more frequent stimulation for a given event facilitates easier transfer from STM to LTM.

Furthermore, it is noteworthy that the BP‐C/CNT artificial synapse devices can display synaptic behavior under identical structural electrical stimulation. When subjected to different voltage polarities, the device demonstrates an increase in current under negative voltage and a decrease under positive voltage. This observation validates synaptic potentiation and inhibition, as exhibited in Figure S8a (Supporting Information). The basic functions of synapses can be successfully simulated, as demonstrated in Figure S8b (Supporting Information) through dual‐pulse heterosynaptic simulation testing (−1 V, 200 ms, *V*_read_ = 0.1 V). The paired‐pulse facilitation (PPF) index in Figure S8b (Supporting Information) can be fitted using the equation *y* = y_0_ + C_1_exp(‐Δt/τ_1_) + C_2_exp(−Δt/τ_2_) with corresponding results presented in Figure S8b (Supporting Information). With increased external stimulation intensity, the EPSC exhibits a rise proportional to pulse amplitude (Figure S8c, Supporting Information), pulse rate (Figure S8d, Supporting Information), pulse width (Figure S8e, Supporting Information), and pulse number (Figure S8f, Supporting Information).

### 2.4 Unisensory Olfactory Response of the Device

**Figure 5:**
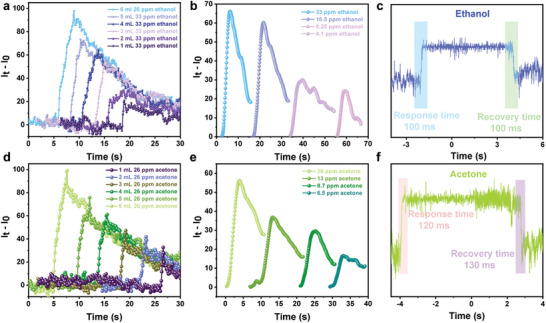
a) Device detection of ethanol gas at different volumes while maintaining the same concentration. b) Device detection of ethanol gas at different concentrations within a concentration. c) Response time to ethanol (33 ppm). d) Device detection of acetone gas at varying volumes with consistent concentration. e) Device assessment of different concentrations of acetone gas in a fixed volume. f) Response time to acetone (26 ppm).

The human olfactory system comprises olfactory receptor neurons, projection neurons, and interneurons that perform remarkably sophisticated functions, including sensing, filtering, memorizing, and forgetting chemical stimuli for perception.^[^ ^31^ ^]^ Developing an artificial olfactory system capable of mimicking these functions has proved to be a challenging task.^[^ ^32^, ^33^ ^]^ In order to conduct olfactory biomimetic simulation, the device structure is optimized by reducing the area of the top ITO and FET, thereby maximizing the contact area between BP‐C/CNT and gas molecules. Ethanol and acetone are chosen as test gases due to their widespread use in laboratory settings, enabling effective management and control of missions for safety and health in production and working environments. Comprehensive test results indicate a decrease in the device's current under ethanol and acetone gas conditions. The device undergoes testing using ethanol gas ranging from 1 to 6 mL at a concentration of 33 ppm, as illustrated in **Figure** **5**a. It is observed that an increase in gas volume corresponds to an augmented change in current. Subsequently, the device's responses to the gas pulse at various concentrations are examined, as appeared in Figure 5b. It is evident that higher ethanol concentrations result in greater changes in current, reflecting enhanced synaptic properties of the device that are compatible with the response of the human olfactory organ. Another crucial parameter for simulating olfactory synapses is the speed of the device's response to the gas, where a faster response aligns more closely with biological synaptic properties. The response and recovery time of the device to ethanol gas are also tested under passive conditions, revealing a rapid response time of ≈100 ms (Figure 5c). Notably, this response time surpasses the fastest sensors reported in existing literature, which typically requires several seconds to achieve similar results. The device exhibits robust gas sensitivity and an ultra‐fast response and recovery time. Employing an identical test methodology for acetone gas yields comparable results, with an increase in current value corresponding to increasing volumes of gas (refer to Figure 5d). Moreover, with the increase in gas concentration, there is a corresponding amplification in the change of current value (Figure 5e). It is worth noting that the response time for acetone exhibits a slightly longer duration compared to ethanol, measuring ≈130 ms (Figure 5f).

### 2.5 Multisensory Optical Olfactory Co‐Sensing

**Figure 6:**
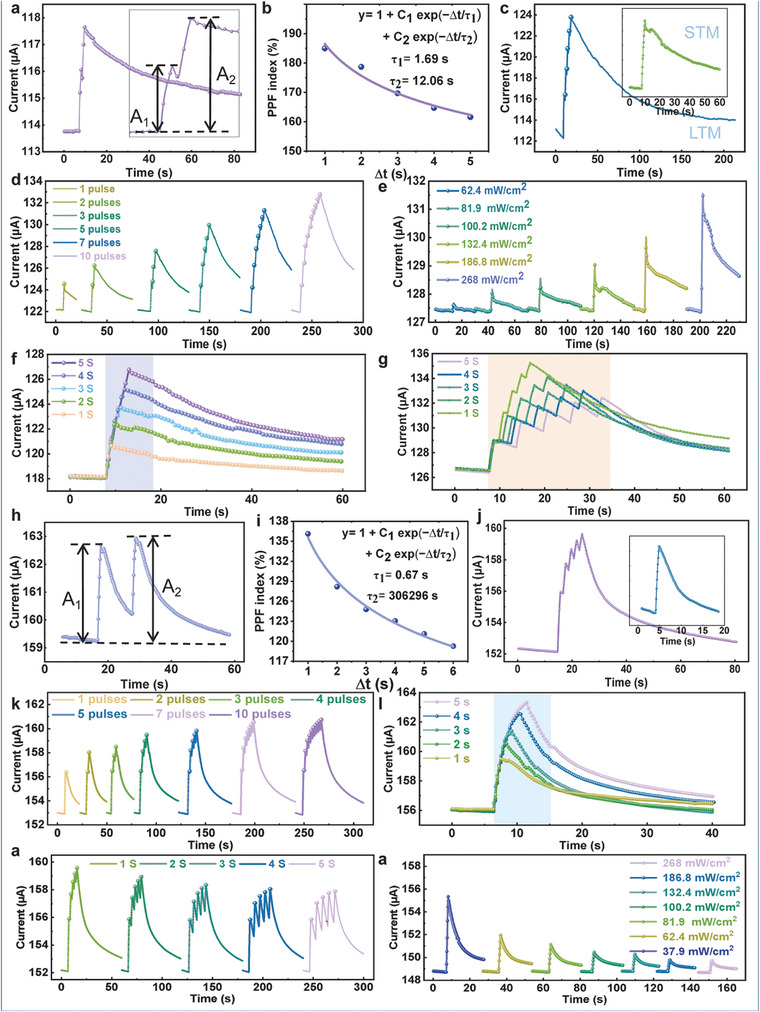
a) Simulation tests of double pulse heterogenization of the device under ethanol gas (365 nm, 286 mW cm^−2^, pulse width 1 s, pulse interval 1 s). b) Ratio of paired‐pulse facilitation index under ethanol gas to the time interval between two pulses. c) The device transitions to LTM mode after five consecutive light pulses under ethanol gas (inset shows STM mode after a single light pulse). d) SNDP tests under ethanol gas under varying numbers of light pulses. e) Peak power dependent plasticity (PPDP) under ethanol gas. f) SDDP tests under ethanol gas under varying light time. g) SRDP tests at different pulse rates under ethanol gas. h) Simulation tests of double‐pulse anisotropy of the device under acetone gas (365 nm, 286 mW cm^−2^, pulse width 1 s, pulse interval 1 s). i) The ratio of the PPF index under acetone gas to the time interval between two pulses. j) After five consecutive light pulses under acetone gas, the device transitions to LTM mode (inset shows STM mode after a single light pulse). k) SNDP tests under acetone gas at different numbers of light pulses. l) PPDP under acetone gas. m) SDDP tests under acetone gas at different light durations. n) SRDP tests at different pulse rates under acetone gas.

By introducing light as a modulating pulse and situating the device within a gaseous environment to emulate olfactory perception, the device successfully mimic the basic fundamental biological synapses, thereby achieving optical olfactory synaptic co‐sensory simulation. A 365 nm light is employed as a modulation pulse to simulate visual perception for visual synaptic function simulation. The device is placed in a gas environment to replicate olfactory perception and imitate olfactory synaptic function. In order to demonstrate synaptic plasticity, an initial simulation is conducted in an ethanol gas environment at an 8 ppm concentration, where two consecutive light pulses are applied (365 nm, 286 mW cm^−2^, pulse width 1 s, pulse interval 1 s). Notably, the second pulse elicits an EPSC that surpasses significantly the response generated by the first pulse (**Figure** **6**a). Typically, facilitatory effects are quantified using the PPF index (A_2_/A_1_ × 100%), wherein *A*_1_ and *A*_2_ represent the amplitude of the response currents elicited by the first and second pulses, respectively. The amplitude of PPF resulting from the second light pulse is determined by the temporal interval between the signals. As the time interval (Δt) between two consecutive pulses increases, the facilitatory effect weakens and there is a decrease in synaptic weight changes (Figure 6b). These experimental PPF indices can be fitted with a double‐exponential function given by *y* = y_0_ + C_1_exp(−∆t/τ_1_) + C_2_exp(−∆t/τ_2_). The fitting results show that τ_1_ and τ_2_ are 1.69 and 12.06 s, respectively. Frequent synaptic stimulation can induce synaptic memory formation and facilitate the transition from STM to LTM, which is another important function of biological synapses. Figure 6c illustrates the current‐time response, depicting a distinct peak in current resulting from a single short light pulse, followed by a return to one‐third of the response current within 50 s, thereby demonstrating STM behavior. When the device receives five consecutive light pulses of random duration, the current returns to one‐third of the response current within 200 s, indicating LTM behavior. This observation underscores the transition from STM to LTM as a consequence of multiple light pulse applications. Effective achievement of the transition from STP to LTP can be accomplished by fine‐tuning optical stimulation parameters, including power density, pulse width, number, and frequency. Simultaneously, during the transition from STP to LTP, there is an increase in both decay time (τ) of the postsynaptic current and the steady‐state current. Notably, with an increase in the number of light pulses (365 nm, 286 mW cm^−2^, pulse width 1 s, pulse interval 1 s), the synaptically generated EPSC value gradually increases, as depicted in Figure 6d.

When the BP‐C/CNT artificial synaptic device is illuminated with optical pulses of different optical power densities (62.4–286.2 mW cm^−2^), the EPSC exhibits a corresponding increase with the rise in optical power density, as illustrated in Figure 6e. Furthermore, the data presented in Figure 6f clearly indicates an increase in EPSC values when the device is stimulated with optical pulses of varying durations (365 nm, 286 mW cm^−2^, pulse widths ranging from 1 to 5 s). In Figure 6g, the EPSC gradually decreases when the device is exposed to continuous light pulses with varying pulse intervals (365 nm, 286 mW cm^−2^, pulse width ranging from 1 to 5 s) over five cycles, as presented. These findings underscore the intricate relationship between optical stimulation parameters and resulting EPSC values, revealing the dynamic behavior of the BP‐C/CNT synaptic device. Subsequently, the same tests are conducted within an acetone gas environment, where successful simulation of basic synaptic properties o is achieved (Figure 6h–n).

**Figure 7:**
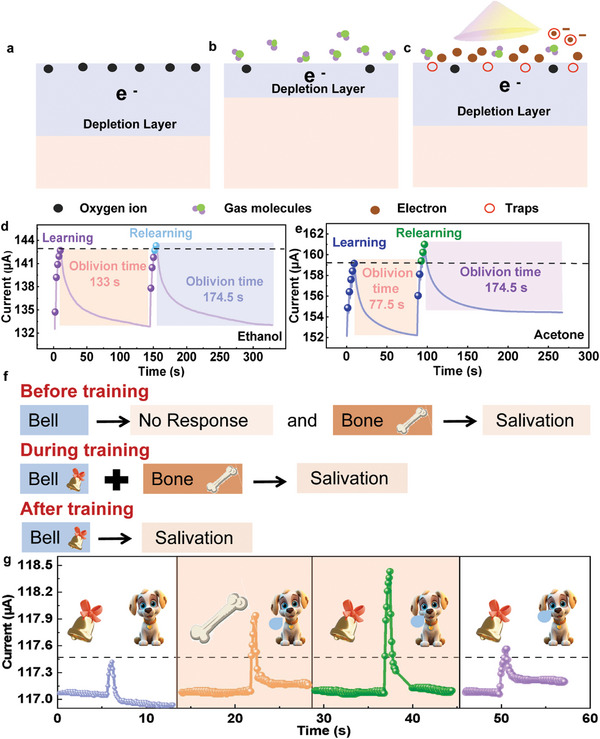
a) The BP‐C/CNT surface state in the air. b) The BP‐C/CNT surface state in the ethanol gas. c) The BP‐C/CNT surface state in the ethanol gas during light exposure. d) BP‐C/CNT‐based synaptic device simulates the “learn‐forget‐learn” function in ethanol gas. e) A BP‐C/CNT synaptic device is used to simulate the “learn‐forget‐relearn” function in acetone gas. f) Pavlov experiment diagram, and g) Simulation of Pavlovian conditioning experiments using synaptic device.

In order to understand the working mechanism of the BP‐C/CNT optical olfactory artificial synaptic co‐sensing, we conducted a comprehensive analysis of device operation in conjunction with first‐principles calculations. The exceptional specific surface area of BP‐C/CNT facilitates the conversion of physically adsorbed O_2_ on the surface of the detector into chemically adsorbed O_2_^−^, O_2_^2−^, O^2−^ etc., resulting in the formation of a space charge depletion layer that reduces electrons in the material conduction band and elevates the surface barrier (**Figure** **7**a). When exposed to ethanol gas, molecules are readily adsorbed onto the BP‐C/CNT surface due to its excellent specific surface area and surface‐active sites. The adsorbed ethanol gas undergoes dehydrogenation reaction with oxygen negative ions on the material surface, leading to acetaldehyde generation. This process generates electrons which transfer the material body, causing alterations in both surface barrier and electron concentration within it (Figure 7b). Subsequently, when light is incident upon the device in the gas environment, numerous photo‐generated carriers are produced on the BP‐C/CNT surface owing to the photoelectric effect of BP‐C/CNT. These photo‐generated carriers can be captured by surface traps, supplementing the internal charge, and inducing changes in the electron concentration (Figure 7c).

The capacity for learning in organisms serves as a significant indicator of their cognitive prowess, referring to the process of associating their own reactions with environmental events,^[^ ^34^ ^]^ and generating meaningful associations between two independent behaviors. The process of encoding and retaining information in biological synapses within the human brain begins with the establishment of STM upon initial data input. Through retraining, the STM can be strengthened, leading to its transformation into LTM. Without such reinforcement, the information would swiftly fade from memory over time. The developed BP‐C/CNT devices can successfully simulate the “learning‐forgotten‐learning” process of experiential learning behavior in the human brain, particularly when subjected to the combined influence of light and smell. The “learning process” is simulated by the continuous application of light pulses to the device in a gaseous environment, increasing the “synaptic weight” by a series of light pulse stimulations, while the forgetting process is simulated by reducing the “synaptic weight” after the continuous light pulse stimulation. Following stimulation, the “synaptic weights” are then elevated once again, thus presenting the process of relearning and memory recovery observed in the human brain. Following the administration of five consecutive light pulses (365 nm, 286 mW cm^−2^, 1 s pulse width, 1 s pulse interval) in the presence of ethanol gas, the conductance can be observed to reach the desired memory level, as depicted in Figure 7d. Subsequently, after the forgetting process lasting for 133 s, four light pulses are required to attain the same synaptic weight level as achieved during the first learning process. Upon administering 10 consecutive light pulses (365 nm, 286 mW cm^−2^, 1 s pulse width, 1 s pulse interval) in an acetone gas environment, it is observed that the conductance achieves the desired memory level (Figure 7e). Subsequently, after the forgetting process lasting for 80.46 s, five light pulses are needed to restore the synaptic weight level equivalent to that obtained during initial learning. In contrast to ethanol, the devices exhibit accelerated learning capabilities in an acetone environment, display prolonged periods of retention, and demonstrate heightened learning efficiencies that facilitate the conversion from STM to LTM conversion, aligning more closely with STM‐LTM processing.

Associative learning is a fundamental cognitive function in the human brain's nervous system, playing a crucial role in the cognitive processes of biological systems.^[^ ^35^ ^]^ This mechanism enables the association of diverse memories with specific events, allowing for the correlation of disparate behaviors. Pavlov's conditioned reflex experiment, illustrated in Figure 7f, serves as a classic illustration of associative learning, where initially an unconditioned response to a bell is transformed into a conditioned response through repeated pairing with food. Subsequently, even in the absence of the food, the dog continues to salivate upon hearing the bell, indicating the establishment of an associative link between the bell stimulus and salivary response. In Figure 7g, Pavlovian conditioned reflex experiments are conducted to examine the association between light exposure and olfaction using the BP‐C/CNT synaptic device as an analog for the dog, substituting light pulse signals for the bell and the acetone gas pulse signal for the bone. The EPSC value for saliva secretion is set at 4 µA. Notably, when light stimulation is applied to the BP‐C/CNT synaptic device in an air environment (without salivating), no significant response is observed. However, upon stimulation with acetone gas pulses, a marked synaptic response is elicited (inducing dog salivating). The concurrent application of light pulses and acetone gas pulses during device training leads to enhanced responses that surpass the threshold. Following training (ringing), the response to light stimuli alone exceeds the threshold. Ultimately, after conditioning, any form of stimulus, whether visual or auditory, could elicit a salivation response, indicating the successful establishment of an associative link between the light pulse and gas pulse signals, effectively simulating the Pavlovian conditioned reflex experiment. Clearly, the current experiments provide a promising alternative for achieving biosensing and memory associations.

## 3 Conclusion

In summary, highly promising carbon doping BP hybrid materials were successfully developed with enhanced environmental stability through an improved chemical vapor transport approach for in situ growth of BP using solid polystyrene as the carbon source and doping source. The improved stability can be attributed to the effective suppression of the oxidation process by C doping, as evidenced by the first‐principles calculations. These devices not only emulate basic synaptic functions under electrical and optical stimuli but also demonstrate exceptional sensitivity and ultra‐low detection limits for ethanol and acetone, with ethanol detection achieving a remarkable response time of 100 ms, surpassing the fastest sensors reported in the existing literature. The resulting devices possess the ability to mimic characteristic features and functionalities of multisensory neurons by simultaneously integrating light modulation, gas sensing, and synaptic functions within a single device. They effectively simulate synaptic functions under different gas conditions, successfully emulating double‐pulse potentiation that facilitates the transition from STM to LTM, while also simulating synaptic learning rules. Furthermore, the devices successfully realize Pavlovian simulation for learning functions. This work could pave the way for widespread application of antioxidant BP materials in optoelectronics and provide a new approach toward studying opto‐olfactory synapse devices—an important step toward flexible and neuromorphic electronics compatible with human physiology.

## 4 Experimental Section

### Synthesis of Carbon Doping BP Material

The carbon doping BP materials were synthesized by the improved chemical vapor transport approach using amorphous red phosphorus as the phosphorus source, tin, and iodine as the transporting agents. Red phosphorus powders (Smart elements, 99.998%) were used as the starting material and ground by using a mortar and pestle. Polystyrene was used as the solid‐state carbon source and doping source. Then weighed red phosphorus: tin powder (Sn, 99.99%): iodine granule (I_2_, 99.8%): polystyrene ratio (PS, 99.9%) of 400: 40: 20: 1 sample into a 1 cm diameter quartz tube for vacuum sealing. Subsequently, the sealed tubes were heated to 650 °C within 200 min in a tube furnace and then held at this temperature for an additional duration of 300 min. Afterward, the sealed tubes were further cooled to 485 °C over the course of 480 min and maintained at this temperature for another period of 240 min for the growth of BP‐C material, followed by a natural cooling to room temperature. The BP crystals, obtained from the sealed tubes, underwent a thorough washing process using hot toluene (≈60 °C), followed by drying under a N_2_ atmosphere, and subsequent storage at ambient conditions for further utilization.

### Synthesis of BP‐C/CNT 2D/1D Heterostructures

Typically, a simple two‐electrode setup in a 50 mL beaker was utilized to exfoliate bulk BP‐C crystals with the cathode consisting of bulk BP‐C (≈50−100 mg) and a Pt mesh used as the anode. The electrolyte was prepared by adding 0.6 g of tetra‐n‐butylammonium acetate (TBA‐CH_3_COO) to an anhydrous, deoxygenated DMF solution (30 mL) for use in the electrochemical exfoliation process. In order to exfoliate bulk BP‐C, a voltage of ≈20 V versus the anode was applied between the Pt anode and BP‐C cathode to initiate the exfoliation process, which was maintained for 2 min, while transforming into a dark‐colored dispersion. Subsequently, sonicated for 5 min produced a dispersion of a few layers of BP‐C flakes suitable for further applications. The DMF solution was gradually replaced with ethanol solution and centrifuged at 8000 r min^−1^ before being spun at room temperature for half an hour to retain only the bottom solution and precipitate. Ethanol was then added to the test tube followed by sonicated for 5 min, and continued centrifugation for another half an hour. This experimental step was repeated three to four times. Finally, coupled BP NSs with CNTs through P─C and C─C bonds resulted in efficient metal‐free BP‐C/CNT (2D/1D) heterostructures by the ultrasonication process.

### Fabrication of PET/ITO/BP‐C/CNT/ITO/PET Synaptic Devices

The BP‐C/CNT mixture solution was continuously pumped and filtered using a vacuum filtration setup to produce a filter film of specific thickness. Subsequently, the film underwent vacuum drying for 12 h. Following the drying process, the prepared film was transferred onto a glass surface, and the filter membrane was removed using an acetone solution. The film was then subjected to an additional 3 h of vacuum drying. Upon completion of the drying process, the prepared thin film was affixed to the PET/ITO substrates using a conductive tape to fabricate PET/ITO/BP‐C/CNT/ITO/PET synaptic devices. Finally, a copper electrode was connected between the conductive tape and the ITO for testing purposes.

### First‐Principles Calculations

The first‐principles calculations were carried out within the frame of density functional theory (DFT), as implemented in Quantum ESPRESSO. The projector augmented wave (PAW) method was used for describing electron‐ion interactions together with exchange‐correlation functional by the generalized gradient approximation (GGA) according to Perdew–Burke–Ernzerhof (PBE). To avoid interaction between the adjacent layers in all supercells, a 20 Å vacuum layer was adopted. The cut‐off energy of 520 eV was employed with convergence thresholds set at values of 10^−5^ eV for energy and 0.02 eV Å^−1^ for force. The first Brillouin zone was sampled using a 3 × 4 × 1 k‐point mesh with a gamma‐centered grid.

### Characterizations

The obtained materials were characterized as follows using field emission scanning electron microscopy (FESEM, S‐4800, Hitachi, Japan), transmission electron microscopy (TEM, Tecnai G2 F20, FEI, USA), double spherical aberration‐corrected transmission electron microscope (ACTEM, JEM GRAND ARM 300F, JEOL, Japan), X‐ray photoelectron spectrometer (XPS, Axis Supra, Shimadzu, UK), and X‐ray diffraction (XRD, Smart Lab 9 kW, Hitachi, Japan), respectively. Electrical and optoelectronic measurements were performed by using a four‐probe station and a semiconductor characterization system (Keithley 4200CSC).

## Conflict of Interest

The authors declare no conflict of interest.

## Author Contributions

L.‐Y.D. and B.‐J.X. contributed equally to this work and co‐first author. L.‐Y.D. carried out the experiments, analyzed the data, plotted and wrote the draft of the manuscript. B.‐J.X. contributed to writing of the manuscript and assisting with the experiment and analysis. S.Y. was involved in guiding the experiment. M.C. participated in some experiments. Y.L. was participated in some experiments. Y.S. was participated in some experiments. Y.N. was participated in some experiments. B.‐S.X. made changes to the manuscript. P.W. carried out the first‐principles calculations. All authors have given approval to the final version of the manuscript. G.‐D.W. supervised this work team.

## Supporting information

Supporting Information

## Data Availability

The data that support the findings of this study are available from the corresponding author upon reasonable request.
