# Supplementary material for: Highly Promising 2D/1D BP‐C/CNT Bionic Opto‐Olfactory Co‐Sensory Artificial Synapses for Multisensory Integration
Source: Adv Sci (Weinh). 2024 Jun 3;11(29):2403665. doi: 10.1002/advs.202403665 (PMC11304314; doi:10.1002/advs.202403665)
Supplement: Supplementary file 1 — Supporting Information [file ADVS-11-2403665-s001.docx]

Supporting Information

Highly promising 2D/1D BP-C/CNT bionic opto-olfactory co-sensory artificial synapses for multisensory integration

*Liyan Dong**^1, ‡^, Baojing Xue^1, ‡^, Guodong Wei**^1,2^*, Shuai Yuan^1^, Mi Chen^1^, Yue Liu^1^, Ying Su^1^, Yong Niu^1^, Bingshe Xu^1,2^, Pan Wang^1^**

1. Y. Dong, B. Xue, G. Wei, S. Yuan, M.Chen, Y. Liu, Y. Su, Y. Niu, B. Xu, P.Wang

^1^ Xi 'an Key Laboratory of Compound Semiconductor Materials and Devices, School of Physics & Information Science, Shaanxi University of Science and Technology, Xi’an 710021, P. R. China.

^2^ Shanxi-Zheda Institute of Advanced Materials and Chemical Engineering, Taiyuan 030024, P. R. China.

E-mail: wgd588@163.com, and wangpan@sust.edu.cn

**Supplementary Figures：**


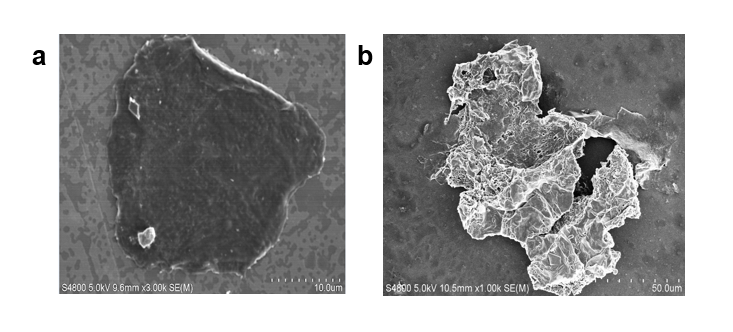


**Figure S1.** (a, b) Typical SEM images of exfoliated 2D BP-C nanosheets.


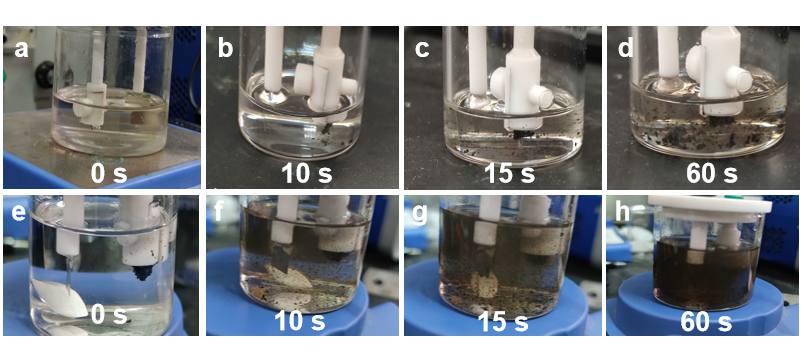


**Figure S2.** (a-d) 2D BP nanosheets stripping process. (e-h) 2D BP-C nanosheets stripping process.


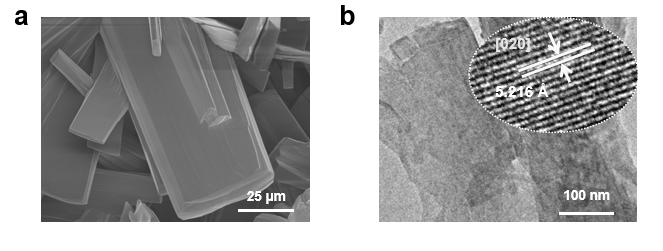


**Figure S3.** (a) SEM image of bulk BP without carbon doping. (b) TEM and its corresponding HRTEM (inset) images of 2D BP nanosheets.


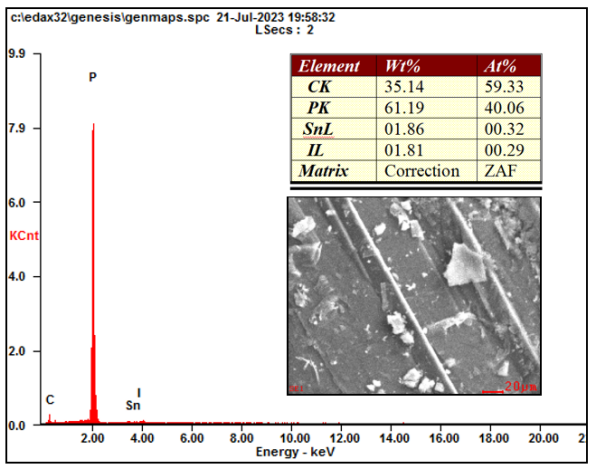


**Figure S4.** Energy spectrum of BP-C material.


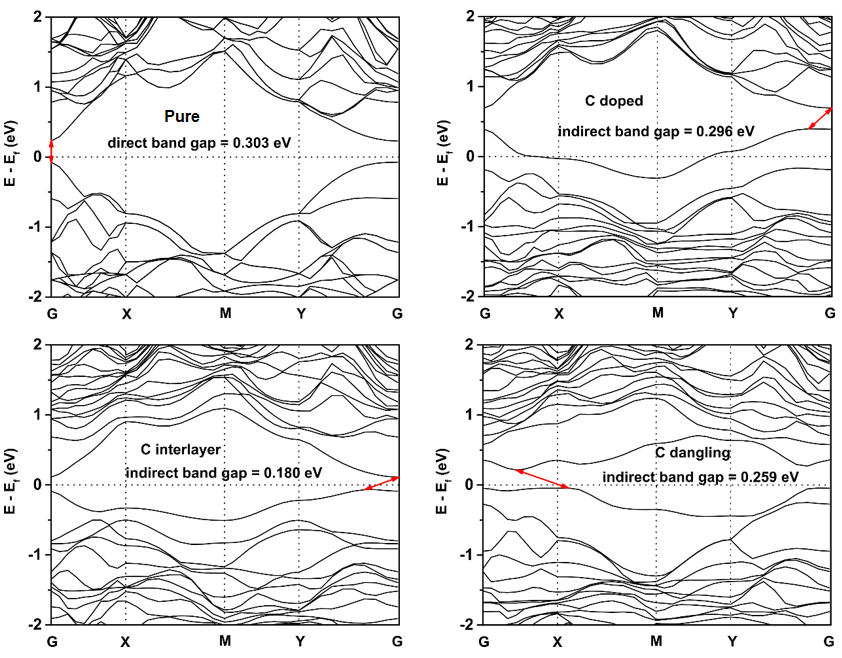


**Figure S5**. The electronic band structure of (a) the ideal bilayer BP and with (b) one P atom replaced by one C atom, (c) one interlayer C atom, and (d) one surface dangling C atom, respectively.


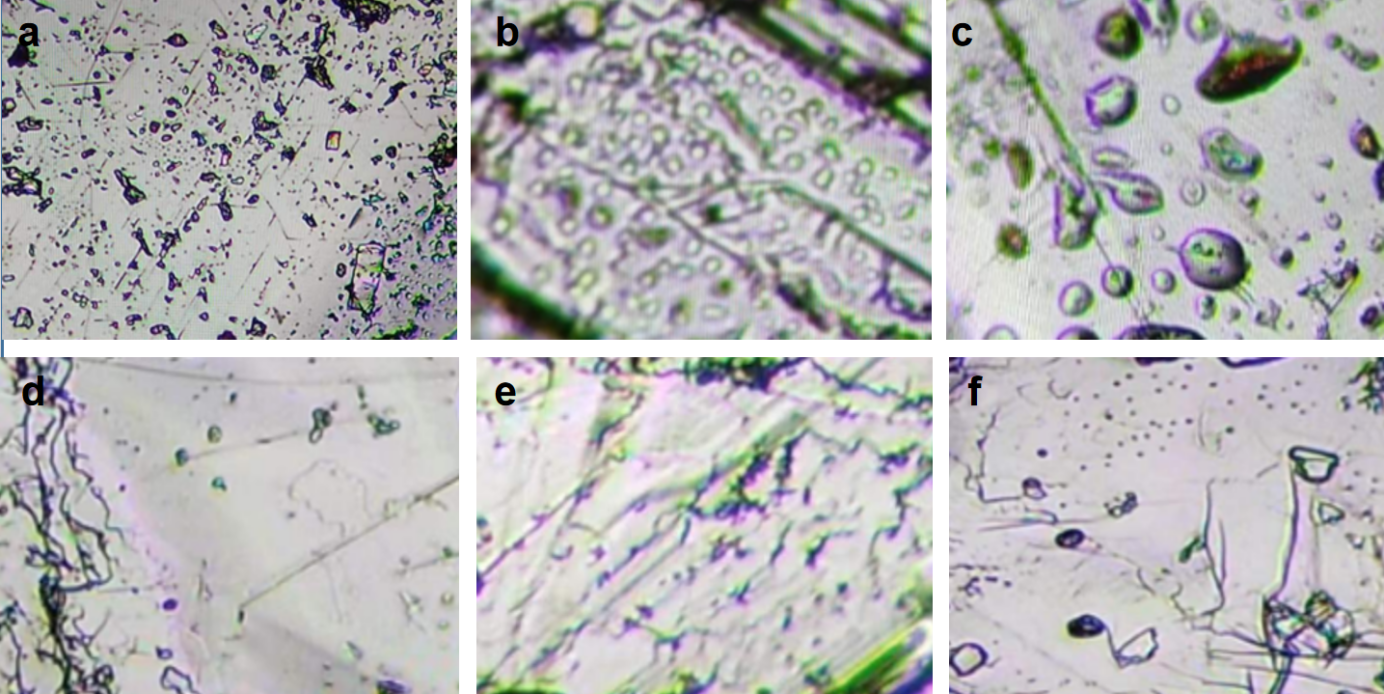


**Figure S6.** Photomicrographs of BP exposure in the air for (a) one day, (b) three months, and (c) one year. Photomicrographs of BP-C exposure in the air for (d) one day, (e) three months, and (f) one year.


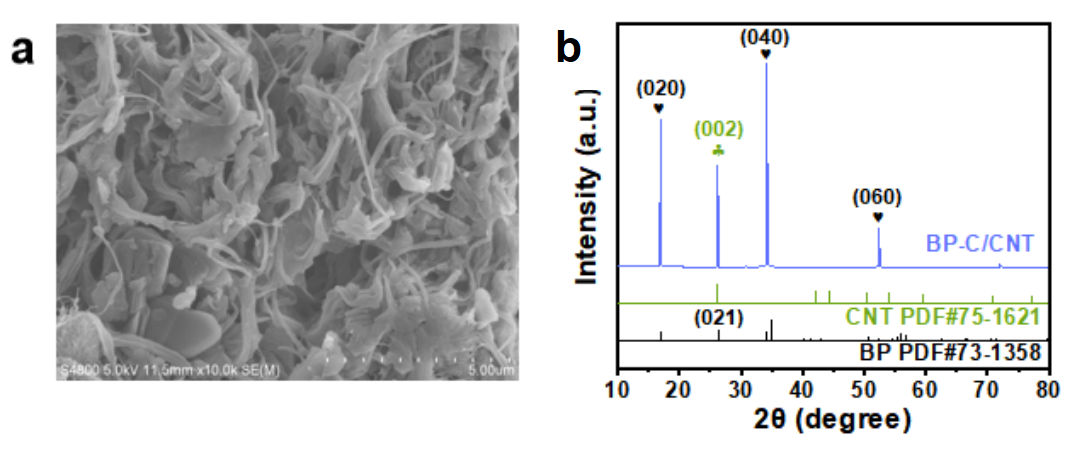


**Figure S7.** (a) SEM image of BP-C/CNT. (b) XRD of BP-C/CNT.


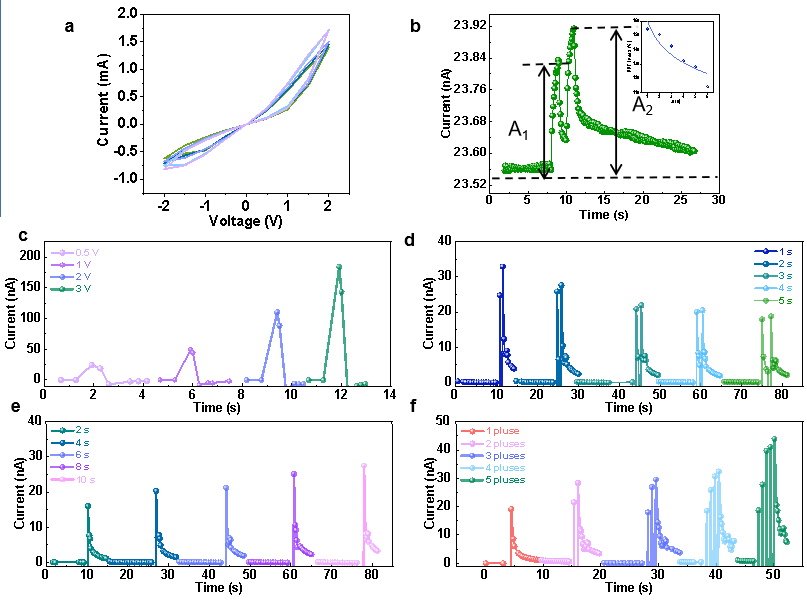


**Figure S8.** (a) Five I-V cycles of the device. (b) Simulation test of the device for two-pulse anisotropy (-1 V, 200 ms, Vread = 0.1 V), the inset shows the ratio of PPF index to the time interval between two pulses. (c) SVDP tests for different pulse amplitudes. (d) SRDP tests for different pulse rates. (e) SWDP tests for different pulse widths. (f) SNDP tests for different pulse counts.


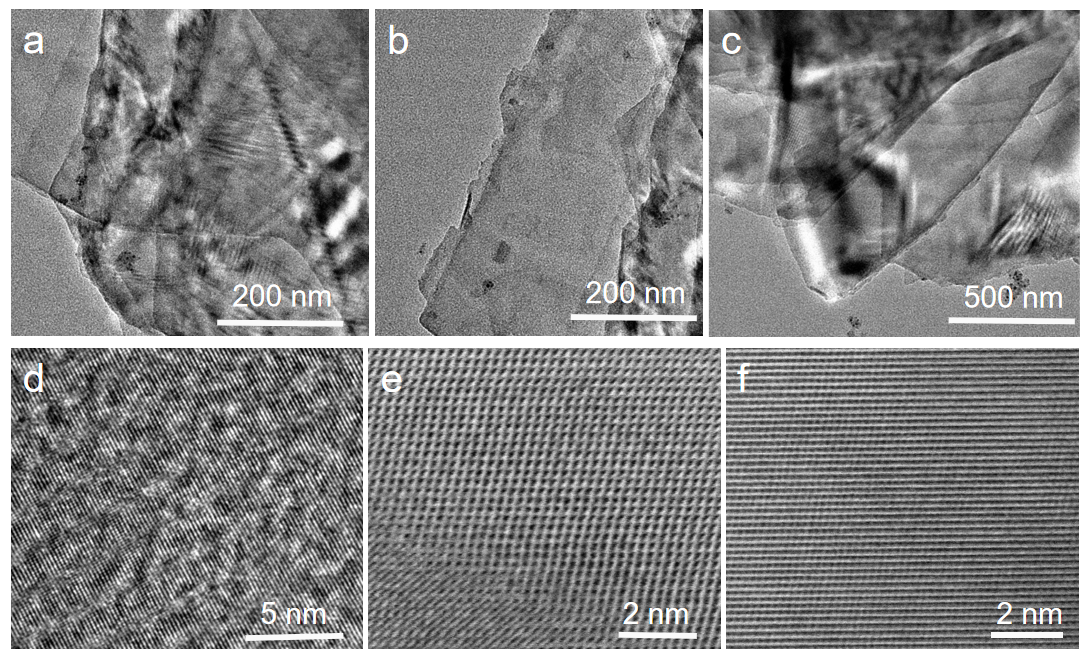


**Figure S9.** (a-c) The typical TEM characterization plots of BP-C. (d) The typical HRTEM characterization plots of BP-C. (e, f) The typical HRTEM characterization plots of BP-C.


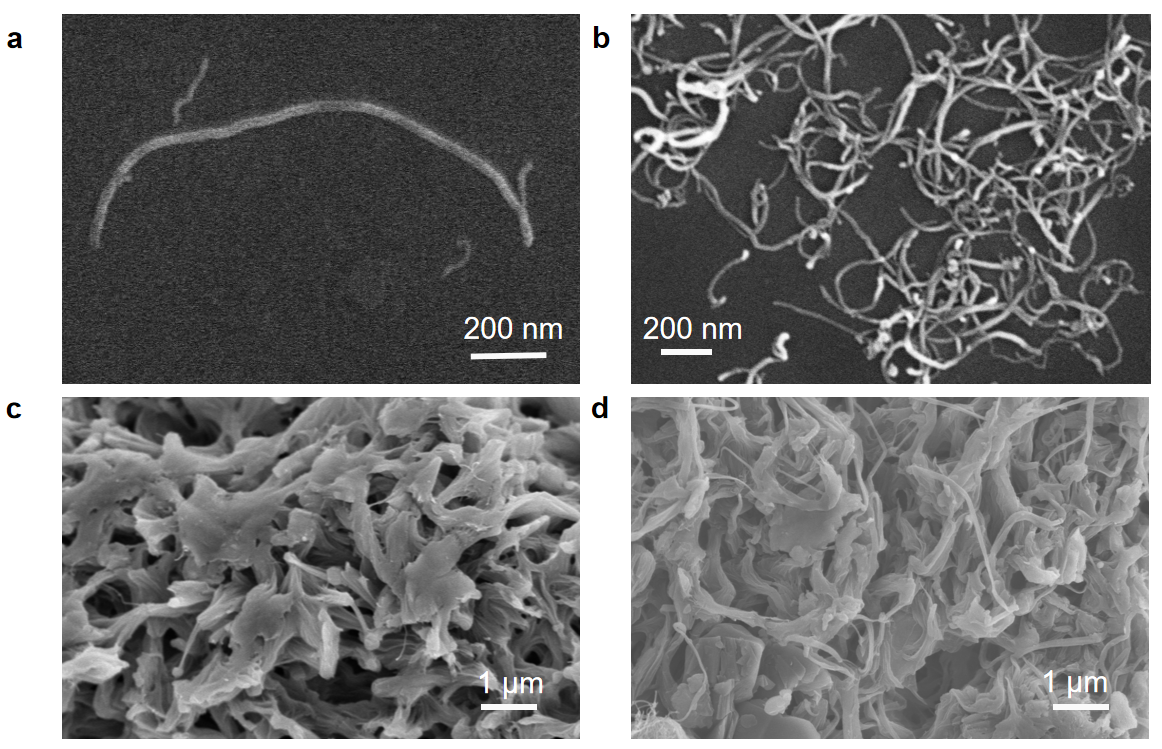


**Figure S10.** (a, b) SEM characterisations of CNTs; (c, d) SEM characterisations of BP-C/CNT membrane.


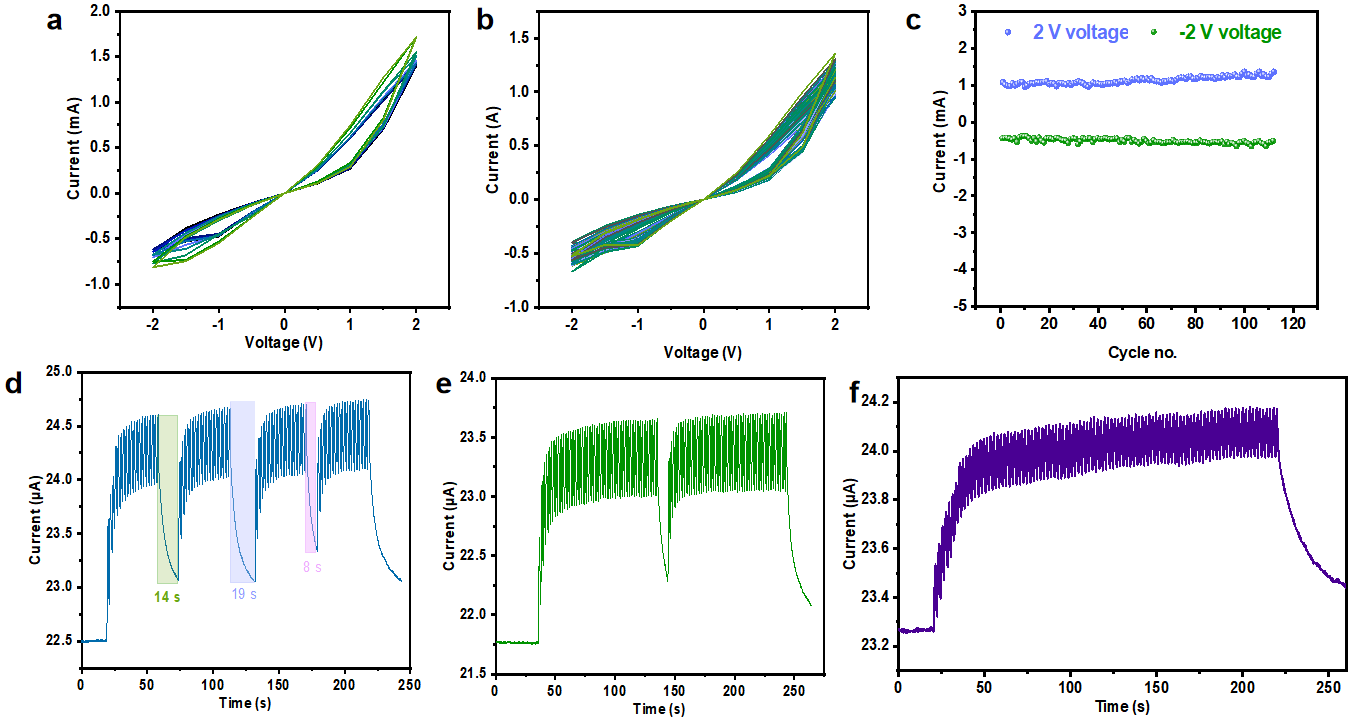


**Figure S11.** (a) 10 I-V cycles for the device. (b) 100 I-V cycles for the device. (c) Current variation of the synaptic device over 100 I-V cycles. (d) 20 consecutive light-pulse tests. (e) 50 consecutive light-pulse tests. (f) 100 consecutive light-pulse tests.


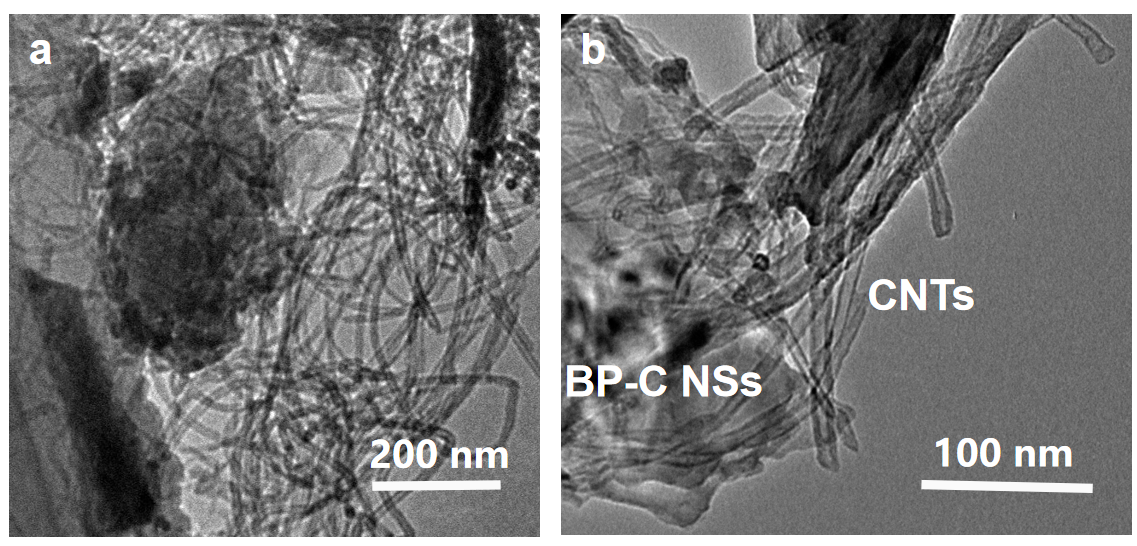


**Figure S12.** (a, b) TEM characterisation of BP-C/CNT membrane.


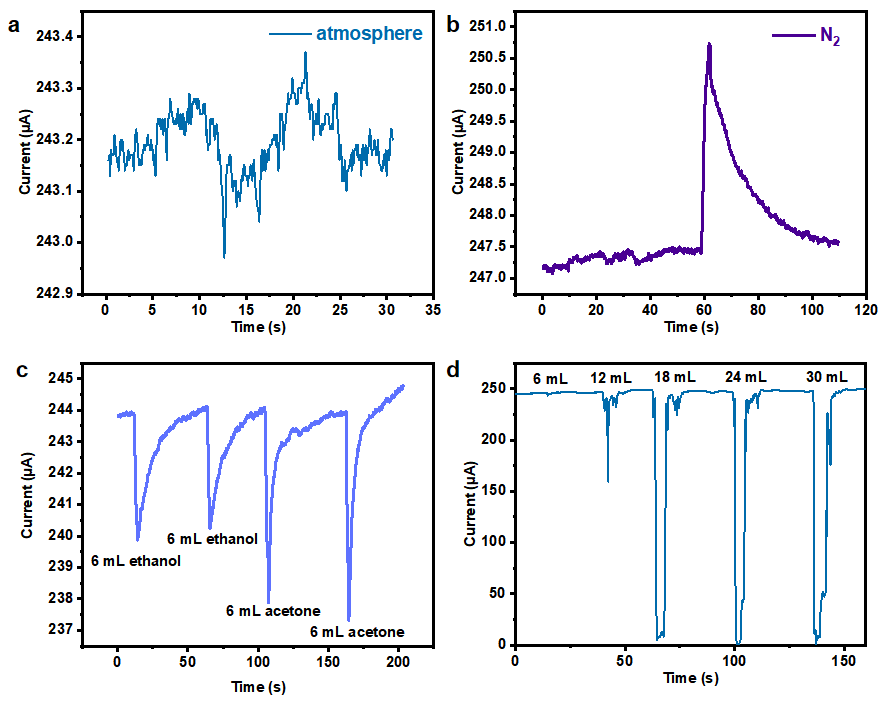


Figure S13. (a) Device detection of the air. (b) Device for the detection of nitrogen gas. (c) Dynamic response of 6 ml of ethanol acetone gas at a concentration of 40 ppm. (d) Dynamic detection of 6 mL, 12 mL, 18 mL, 24 mL, and 30 mL of ethanol gas at a concentration of 40 ppm.


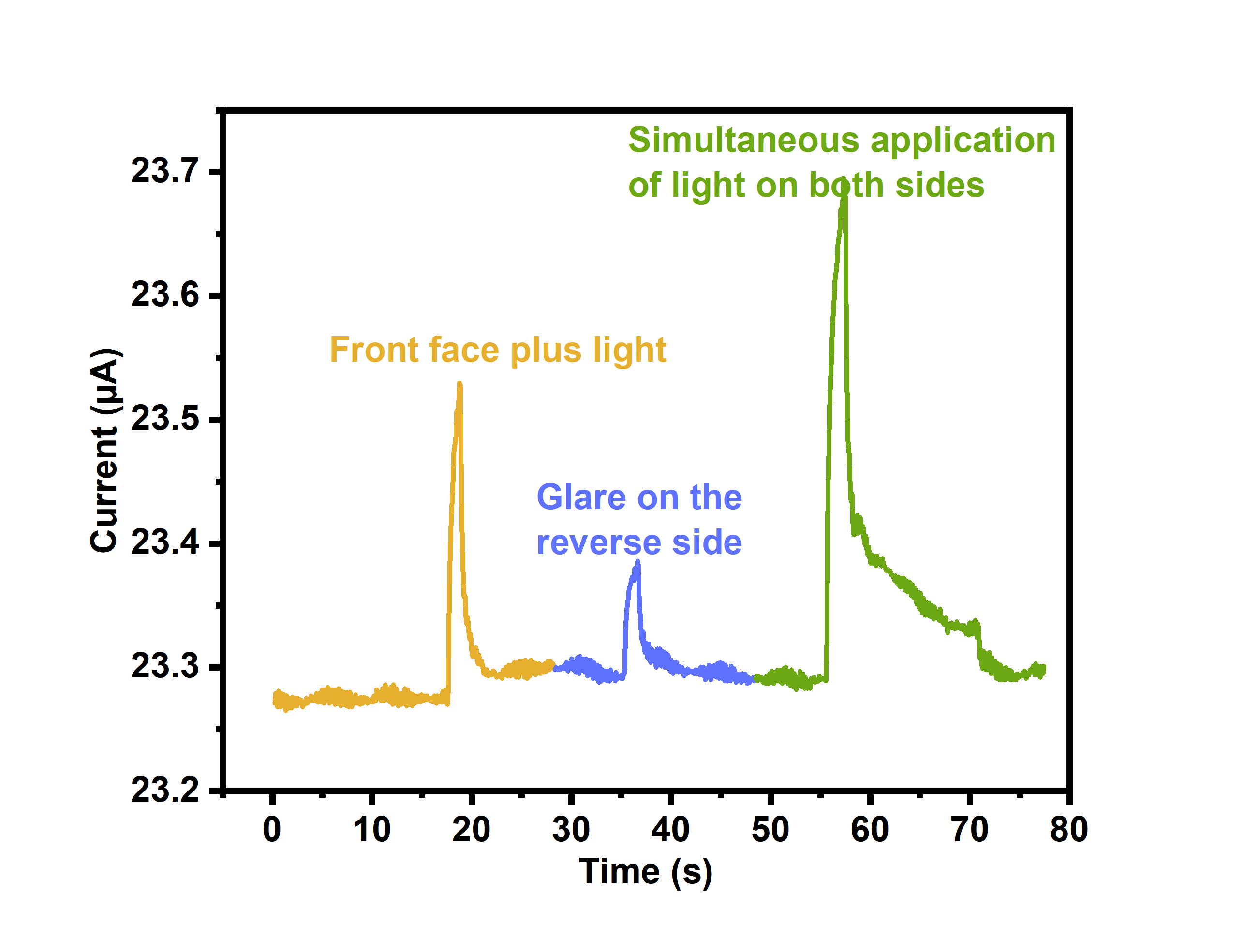


**Figure S14.** Light-pulse stimulation in both directions.

**Supplementary Table：**

**Table S1.** The calculated neutral defect formation energies (E^f^ ) of one P atom replaced by one C atom, one interlayer C atom, and one surface dangling C atom.

| Defect type | Substitution | Interlayer | Dangling |
| --- | --- | --- | --- |
| $\boldsymbol{E}^{\boldsymbol{f}}\mathbf{(eV)}$ | 1.88 | 3.29 | 3.12 |

**Defect formation energies**

The defect formation energies are defined as the equation (1) ^[1,2]^:

$E^{f}=E_{doped\_BP}^{tot}-E_{0}^{tot}+n_{P}\mu_{P}-n_{C}\mu_{C}$ (1)

, where $E_{doped\_BP}^{tot}$ is the total energy of the C-doped BP, $E_{0}^{tot}$ is the total energy of the ideal BP, $n_{P}$ is the number of P atoms being removed, the chemical potential of P atom ($\mu_{p}$) is -5.36 eV calculated from the energy of one P atom in the ideal BP, $n_{C}$ is the number of C atoms being added, and the chemical potential of C atom ($\mu_{C}$) is -9.22 eV calculated from the graphite, respectively.

**Table S2.** Comparison of artificial synapse devices.

| **Artificial synapse device** | **Synaptic behavior** | **Stimuli (mV)** | **Logical operation** | **Multisensory** |
| --- | --- | --- | --- | --- |
| Au/MCA−BP/Au^3^ | Yes | 500 | No | No |
| Ag/N-GOQDs/Pt^4^ | Yes | 100 | No | No |
| Synaptic barristor based on 2D heterostructures^5^ | Yes | ~200 | No | No |
| BP/MoS_2_ transistors^6^ | Yes | 2000 | Yes | No |
| BP based floating gate synaptic^7^ | Yes | 10000 | Yes | No |
| BP charge-trap memory device^8^ | No | 10000 | No | No |
| This work | Yes | 50 | Yes | Yes |

**Table S3.** Gas sensor response time comparison table

| **Gas sensors** | **Ethanol response time** | **Ethanol recovery time** | **Acetone response time** | **Acetone recovery time** |
| --- | --- | --- | --- | --- |
| SnO_2_-ZnO gas sensor^9^ | 3 s | 45 s | 3 s | - |
| SnO_2_ gas sensor^10^ | 16 s | 40 s | - | - |
| ZnFe_2_O_4_ nanosheets gas sensor^11^ | - | - | 23 s | 9 s |
| Bismuth ferrite gas sensor^12^ | - | - | 48 s | 1 min |
| Ba_0.25_Sr_0.25_Fe_2.5_O_4_ gas sensor^13^ | 12 s | 5 s | 11 s | 8 s |
| (La_0.8_Ca_0.2_)_0.6_ Bi_0.4_FeO_3_ gas sensor^14^ | 16 s | 17 s | 1000 | No |
| La_1–2x_Ba_x_Bi_x_FeO3 (0 ≤ x ≤ 0.2)^15^ | 5-10 s | 5-10 s | - | - |
| This work | 0.1 s | 0.1 s | 0.12 s | 0.13 s |

**REFERENCES**

[1] H. Cheng, J. Zhou, M. Yang, L. Shen, J. Linghu, Q. Wu, P. Qian, Y. P. Feng. J. Mater. Chem. C. **2018**, *6*, 8435-8443.

[2] K. Iordanidouc, M. Houssa, J. Kioseoglou, V. V. Afanas’ev, A. Stesmans, C. Persson. ACS Appl. Nano Mater. **2018,** *1*, 6656-6665.

[3] Y. L. Hsieh, C. Y. Su. ACS Applied Nano Materials. **2021**, 4, 9584-9594.

[4] A. S. Sokolov, M. Ali, R. Riaz, Y. Abbas, M. J. Ko, C. J. Choi. Advanced Functional Materials. **2019**, 29, 1807504.

[5] W. Huh, S. Jang, J. Y. Lee, D. Lee, D. Lee, J. M. Lee, H. G. Park, J. C. Kim, H. Y. Jeong, G. Wang. Advanced Materials. **2018**, 30, 1801447.

[6] X. Xiong, J. Kang, Q. Hu, C. Gu, T. Gao, X. Li, Y. J. Wu. Advanced Functional Materials. **2020**, 30, 1909645.

[7] X. Xiong, X. Wang, Q. Hu, X. Li, Y. Wu. Iscience. **2022**, 25, 103947.

[8] H. Tian, B. Deng, M. L. Chin, X. Yan, H. Jiang, S. J. Han, V. Sun, Q. Xia, M. Dubey, F. Xia. ACS nano. **2016**, 10, 10428-10435.

[9] B. Jiang, T. Zhou, L. Zhang, J. Yang, W. Han, Y. Sun, F. Liu, P. Sun, H. Zhang, G. Lu. Sensors and Actuators B: Chemical. 2023, 393, 134257.

[10] M. Souri, Y. Yamini, H. S. Amoli. Materials Science and Engineering: B. 2023, 294, 116501.

[11] Z. Wen, H. Ren, D. Li, X. Lu, S. W. Joo, J. Huang. Sensors and Actuators B: Chemical, 2023, 379, 133287.

[12] P. Ghadage, P. Shinde, K. D. Nadargi, J. Nadargi, H. Shaikh, M. A. Alam, I. Mulla, M. S. Tamboli, J. S. Park, S. Suryavanshi. RSC advances, 2024, 14: 1367-1376.

[13] B. Abdelaoui, A. Benali, M. Bejar, E. Dhahri, M. P. Graça, M. A. Valente, P. Lin, J. Wu. Colloids and Surfaces A: Physicochemical and Engineering Aspects, *2024,* 133789.

[14] A. Benali, E. M. Benali, B. M. G. Melo, A. Tozri, M. Bejar, E. Dhahri, M. P. F. Graca, M. A. Valente, P. Lin, J. Wu, B. F. O. Costa. Journal of Materials Science: Materials in Electronics, **2023**, *34*, 45.

[15] E. M. Benali, A. Benali, M. Bejar, E. Dhahri, V. A. Khomchenko, L. Peng, J. Wu, B. F. O. Costa. Journal of Alloys and Compounds, **2021**, *883,* 160856.
